# Navigating young minds: reliability and validity of the Greek version of kiddie – schedule for affective disorders and schizophrenia – present and lifetime DSM-5 version (K-SADS-PL-GR-5)

**DOI:** 10.1186/s12888-023-05076-1

**Published:** 2023-08-22

**Authors:** Gerasimos Kolaitis, Foivos Zaravinos-Tsakos, Ioannis-Marios Rokas, Ioannis Syros, Antonia Tsakali, Maria Belivanaki, Georgios Giannakopoulos

**Affiliations:** 1 Department of Child and Adolescent Psychiatry, School of Medicine, National and Kapodistrian University of Athens, “Aghia Sophia” Children’s Hospital, Athens, 115 27 Greece; 2 School Of Medicine, National and Kapodistrian University of Athens, Athens, Greece

**Keywords:** K-SADS-PL, Validity, Reliability, DSM-5

## Abstract

**Background:**

The Kiddie-Schedule for Affective Disorders and Schizophrenia-Present and Lifetime Version (K-SADS-PL) is one of the most popular semi-structured psychiatric interviews for children and adolescents. Its latest DSM-5 version (K-SADS-PL DSM-5) has only recently been adapted and validated in various languages. In the present study, we aimed to investigate the reliability and validity of the Greek version of the K-SADS-PL DSM-5.

**Methods:**

A total of 137 patients consecutively referred for admission, aged 7–17, were included. The K-SADS-PL DSM-IV was translated and adapted to correspond to DSM-5 categories. Convergent and divergent validity were assessed against two self-report rating scales, Children’s Depression Inventory (CDI) and Screen for Child Anxiety Related Emotional Disorders (SCARED). Inter-rater reliability was calculated exclusively for instances where a diagnosis involved three or more patients.

**Results:**

Our findings revealed good to excellent inter-rater reliability and good to excellent consensual validity across most psychiatric diagnoses, except for panic disorder. Diagnostic efficiency, measured by sensitivity, specificity, positive and negative predictive values, consistently showed high specificity and negative predictive validity across all diagnostic categories.

**Conclusions:**

These findings support the applicability of the Greek version of the K-SADS-PL DSM-5 as a reliable and valid diagnostic tool in Greek-speaking populations.

## Introduction

The use of structured and semi-structured diagnostic interviews is of paramount importance in child and adolescent psychiatry, as it allows the objective assessment and diagnosis by decreasing sources of variability. Initially designed as research tools, diagnostic interviews are widely used to assess the course of disorders and response to treatment, thereby improving the reliability of clinical information. One of the most commonly used semi-structured psychiatric interviews is the Kiddie Schedule for Affective Disorders and Schizophrenia, Present and Lifetime version (K-SADS-PL) for children and adolescents aged 6–18 years [1]. The K-SADS-PL is designed to correspond to the Diagnostic and Statistical Manual for Mental Disorders fourth edition (DSM-IV) [2], assessing current and past episodes of psychopathology across a broad spectrum of psychiatric diagnoses.

Following the publication of DSM-5 [3], Kaufman et al. [4] modified the K-SADS-PL to adapt to the changes made to the latest edition (K-SADS-PL DSM-5). Specifically, diagnostic categories have been added [disruptive mood dysregulation disorder (DMDD), avoidant restrictive food intake disorder (ARFID) and binge eating disorder (BED)], while others [autism spectrum disorder (ASD), intermittent explosive disorder (IED) and social anxiety disorder (SAD)] suggest modified versions of diagnoses that were already in the DSM-IV. Furthermore, clinical conditions, such as non-suicidal self-injury (NSSI) and limited prosocial emotions (LPE), have also been included.

K-SADS-PL DSM-IV has been translated and adapted into over 20 different languages, with fair-to-excellent reliability and validity. Studies of the Spanish and Hebrew adaptations revealed good inter-rater reliability (κ ≥ 0.6) for most diagnostic categories [5, 6]. As for convergent validity, the Korean and Brazilian-Portuguese version determined significant correlations between K-SADS-PL DSM-IV and Child Behavior Checklist (CBCL) [7, 8]. Furthermore, an Icelandic study partially confirmed the screen criteria of K-SADS-PL DSM IV for major depressive disorder, given the high comorbidity among the sample [9]. Regarding the empirical validation of the K-SADS-PL DSM-5, several studies have been reported. Studies of the Turkish, Spanish and Icelandic K-SADS-PL DSM-5 revealed good reliability (κ ≥ 0.6) and generally good construct validity [10–12]. In concordance with these findings, the Portuguese adaptation of the K-SADS-PL DSM-5 showed fair-to-excellent inter-rater reliability (κ = 0.44–0.93) for all diagnosed disorders [13]. Consensual validity has been examined by Nishiyama et al. [14] for the Japanese version of K-SADS-PL DSM-5. They found good to excellent inter-rated reliability (κ = 0.89–1.00) for all disorders and acceptable consensual validity. Next to the emphasis of comprehensiveness across a wide range of disorders, the K-SADS-PL DSM-5 has showed good reliability and validity on single syndromes such as Attention Deficit-Hyperactivity Disorder (ADHD), Post-Traumatic Stress Disorder (PTSD), and DMDD) [15–17].

To date, studies of the Greek version of the K-SADS-PL DSM-5 are lacking. The only study that addressed psychometric properties is our previous study using the Greek version of the K-SADS-PL [18]. The study revealed excellent inter-rater reliability for depressive (κ = 0,90), anxiety (κ = 0.80) and conduct disorders (κ = 0.90). In the present study, therefore, we investigated the inter-rater reliability, consensual validity, and construct validity of the Greek version of K-SADS-PL DSM-5. This is the first study on the reliability and validity of the K-SADS-PL DSM-5 in Greece.

## Methods

### Participants

The sample consisted of children and adolescents who were consecutively referred for admission to the University Department of Child Psychiatry at “Aghia Sophia” Children’s Hospital, Athens, Greece, between August 2021 and February 2022. The inclusion criteria were: (i) all new referrals aged between 7 and 17 years; and (ii) children/adolescents and/or parents/caregivers who could cooperate to complete the interview. The exclusion criteria were: (i) organic psychiatric disorders; (ii) children/adolescents presented with severe agitation and impulsivity; (iii) children/adolescents with mutism and/or communication impairments; and (iv) parents/caregivers who were unable to complete the interview. Informed consent was obtained from the parents and assent was obtained from the patients. This study was approved by the Institutional Board of “Aghia Sophia” Children’s Hospital and the Ethics Committee of School of Medicine, National and Kapodistrian University of Athens, Greece.

### Measures

#### K-SADS-PL-5

After obtaining permission from the original developers, the K-SADS-PL DSM-5 was modified based on the original English of DSM-5 and the prior Greek version of K-SADS-PL, which was translated and developed by the current authors. To ensure conceptual equivalence with the original version, the Greek version of the K-SADS-PL DSM-5 was developed through a classic translation-back translation technique, following the principals of good practice for the translation and cultural adaptation process for patient-reported outcomes measures [19]. Each module of the K-SADS-PL DSM-5 was translated independently from English to Greek by two child psychiatrists. Both translated versions were compared by the first author who produced a reconciled version. Then, a bilingual Greek-English, blinded to the original versions, back-translated the K-SADS-PL DSM-5 into English. Two expert child psychiatrists reviewed the back-translated revisions and modified the primary version of the K-SADS-PL DSM-5 accordingly. The completed version K-SADS-PL DSM-5 was initially applied at the Department of Child and Adolescent Psychiatry, “Aghia Sophia" Children’s Hospital”, by four child psychiatrists to evaluate its feasibility. After adjusted on their clinical suggestions, the instrument was brought to use in the study.

The K-SADS-PL DSM-5 is a semi-structured diagnostic interview that ascertains current and lifetime diagnoses based on DSM-5 criteria, in children and adolescents aged 6 to 18 years. A trained clinician conducts the interview, gathering information from the parent/caregiver and the child/adolescent to establish a comprehensive range of symptoms and their course. The K-SADS-PL DSM-5 includes 39 disorders, integrated through an introductory interview, screening questions, diagnostic supplements, and a summary lifetime diagnostic checklist. Screening questions encompass key symptoms for each disorder, with various probes and scoring criteria, along with skip-out criteria describing current and past episodes. Key symptoms are coded using 0 (no information available) to 3 (threshold criteria) rating scale. In case of a child/adolescent receives a score of “3” in any of the symptoms assessed through a K-SADS-PL-5 screening interview, the clinician/interviewer will administer the corresponding diagnostic supplement. The diagnostic supplements include (1) depressive and bipolar-related disorders; (2) schizophrenia spectrum and other psychotic disorders; (3) anxiety, obsessive compulsive, and trauma-related disorders; (4) impulse control and disruptive behavior disorders; (5) eating disorders and substance-related disorders; and (6) neurodevelopmental disorders.

#### Children’s Depression Inventory (CDI)

The Children’s Depression Inventory (CDI) is a 27-item self-reported questionnaire assessing symptoms of depression in children and adolescents 7 to 17 years [20]. The CDI is rated on a 3-point Likert scale (0 = absence of symptoms, 1 = mild symptoms, 2 = definite symptoms) quantifying depressed mood, hedonic capacity, vegetative functions, self-evaluation and interpersonal behaviors, within the past two weeks. Total scores range from 0 to 54, whereas higher sum scores indicate higher depressive state. The Greek version of the CDI has demonstrated good psychometric, with a cut-off score of ≥ 15 suggesting probable depression [21].

#### Screen for Child Anxiety Related Emotional Disorders (SCARED)

The Screen for Child Anxiety Related Emotional Disorders (SCARED) is a 41-item self-report measure for screening anxiety symptoms in children and adolescents [22]. The SCARED has yielded five factors: somatic/panic, generalized anxiety, separation anxiety, social phobia, and school phobia. Each item is rated using a 3-point scale (0 = not true or hardly ever true; 1 = sometimes true; 2 = true or often true), on a 3-month period. Total scores range from 0 to 82 with higher scores indicating more severe anxiety symptoms. The SCARED has demonstrated high reliability and validity in both clinical and non-clinical populations [16]. For this study, SCARED demonstrated excellent internal reliability (α = 0.93).

#### Interviewers

All K-SADS-PL DSM-5 interviews were performed by four experienced child and adolescent psychiatrists. Before the interviews, all the interviewers underwent appropriate training regarding the instrument, the diagnostic classification, and the use of the K-SADS-PL DSM-5. The training consisted of a two full day workshop given by an experienced child and adolescent psychiatrist (GK) who had undergone K-SADS-PL DSM-5 training and had been skilled in its operation for more than 15 years. The training course included (i) presentations of structured interviewing in child and adolescent psychiatry and the history of the K-SADS, (ii) reading of the interview, (iii) role-playing by trainees (alternating between interviewer/interviewee), (iv) watching and scoring several video interviews, and (v) administering the K-SADS-PL DSM-5 themselves under the supervision of an experienced clinician.

#### Procedure

An unstructured interview by an experienced child and adolescent psychiatrist who was familiar with the DSM-5 classification and diagnostic criteria was used in clinical intake procedure with every patient and their parents. The unstructured interviews followed standard principles with the aim to ensure that all DSM-5 diagnoses were systematically evaluated. The administration technique involved first the clinical interview for all patients in their first week of admission, before interviews using the K-SADS-PL DSM-5. For the K-SADS-PL DSM-5 patients and parent(s) were interviewed simultaneously for up to 3-hours. After interviewing parent and child, a summary rating was derived from all sources of information available which complimented the clinician’s judgment. To maintain blindness, clinicians performing the clinical interviews were not involved in any procedure regarding the translation, review, and implementation of the K-SADS-PL DSM-5. Both patients and parents were advised not to discuss or share any diagnostic information during the K-SADS-PL DSM-5 interviews. The clinical interviews and K-SADS-PL DSM-5 were an average one week apart. Following the administration of the K-SADS-PL DSM-5, children and adolescents completed the self-report questionnaire regarding their depressive and anxiety symptoms.

To obtain inter-rater reliability, 27 patients were randomly selected and were interviewed by an interviewer-coder pair. The interviews were conducted by the interviewer, according to the initial procedure, while the coder independently completed the diagnostic coding sheet. Furthermore, the coder checked to verify the accurate utilization of threshold criteria and assignment of the final diagnosis. Any discrepancies between interviewer-coder ratings, were resolved through consensus.

### Statistical analysis

The demographic and diagnostic data of the subjects were reported using numbers and percentages for categorical variables, and means, standard deviations and medians (minimum-maximum) for continuous variables. To examine the consensual validity and inter-rater reliability of the K-SADS-PL DSM-5, the Cohen’s kappa statistic was applied. The kappa coefficients were interpreted as excellent (κ > 0.75), good (κ = 0.59–0.75), fair (κ = 0.40–0.58) and poor (κ < 0.4) [23]. Kappa statistic was calculated when the number of each K-SADS-PL DSM-5 diagnosis was ≥ 3. Sensitivity, specificity, positive and negative predictive values were evaluated for the threshold diagnoses of K-SADS-PL DSM-5 using the Chi-square test to compare clinical and K-SADS-PL DSM-5 diagnoses. Convergent validity was examined between the K-SADS-PL DSM-5, CDI and SCARED using point biserial correlation coefficients and effect sizes of differences between cases and non-cases. Effect size (ES) for independent groups was calculated by Cohens’ d (0.2 for small effect size; 0.5 for medium effect size; 0.8 for large effect size) [24]. All reported p values are two-tailed. Statistical significance was set at p < 0.05 and analyses were conducted using SPSS statistical software (version 26.0).

## Results

### Demographic and clinical data/Diagnostic profile

The participants were 137 children and adolescents, aged 7 to 17 years. The median age was 13 years. Sixty-seven patients were male (48.9%) and 70 (51.1%) were female. A total of 49 of the patients (35.7%) had comorbidities, and the number of comorbidities ranged from 1 to 3, based on the K-SADS-PL DSM-5. Twenty-nine patients did not meet criteria for any psychiatric disorder in either K-SADS-PL DSM-5 or the clinical diagnosis. Of the 11 diagnostic areas included in the diagnostic supplements, the most frequently diagnosed disorder was depressive disorder (61.4%), followed by generalized anxiety disorder (25.2%), persistent depressive disorder (14.2%), obsessive compulsive disorder (8.6%) and schizophrenia (8.6%). As shown in Table 1, most of the psychiatric disorders were mood disorders (75.6%) and anxiety disorders (38.6%).

**Table 1:** K-SADS-PL-GR-5 and clinical diagnoses

	K-SADS-PL-5		Clinical diagnosis		Kappa	P value
	N	%	N	%	k	
Mood disorders	96	75.6	65	72.5	0.69	< 0.001
Major Depressive Disorder	78	61.4	56	49.6	0.77	< 0.001
Persistent Depressive Disorder	18	14.2	9	8.0	0.80	< 0.001
Neurodevelopmental disorders and behavior disorders	32	25.2	7	12.3	0.58	< 0.001
Attention Deficit Hyperactivity Disorder	11	8.6	4	3.5	0.65	< 0.001
Conduct Disorder	10	7.8	3	2.7	0.65	< 0.001
Oppositional Defiant Disorder	11	8.6	-	-	-	-
Anxiety disorders	49	38.6	17	25.6	0.66	< 0.001
Generalized Anxiety Disorder	32	25.2	12	10.6	0.74	< 0.001
Separation Anxiety	4	3.1	2	1.8	0.66	< 0.001
Panic Disorder	5	3.9	1	0.1	0.49	< 0.001
Specific Phobia	3	2.3	2	1.8	0.80	< 0.001
Social Anxiety Disorder	5	3.9	-	-	-	-
Obsessive Compulsive Disorder	11	8.6	7	6.2	0.76	< 0.001
Schizophrenia	11	8.6	8	7.1	0.83	< 0.001
Enuresis	6	4.7	12	3.5	0.79	< 0.001

### Consensual validity

Good to excellent consensual validity was found across most psychiatric diagnoses with a range of Cohen’s Kappa from 0.65 to 0.830, but fair for panic disorder (0.49). Kappa values for spectrum diagnoses (mood disorders, neurodevelopmental disorders, anxiety disorders) were good. In addition, despite observed similarity between the most prevalent disorders, all disorders displayed higher prevalence on K-SADS-PL DSM-5 as compared to clinical diagnosis. The frequency of K-SADS-PL DSM-5 and clinical diagnoses and the agreements between them are presented in Table 1.

### Inter-rater reliability

The inter-rater reliability of K-SADS-PL DSM-5 for major depressive disorder, obsessive compulsive disorder, schizophrenia, enuresis, separation anxiety disorder, ADHD and conduct disorder was excellent (0.78–0.84). The inter-rater reliability for generalized anxiety disorder was good (0.73) (Table 2).

**Table 2:** Inter-rater reliability of K-SADS-PL-GR-5

	Interview (N)	Agreement (%)	Kappa (k)	P value
Major Depressive Disorder	18	85	0.84	< 0.001
Generalized Anxiety Disorder	9	88	0.73	< 0.001
Obsessive Compulsive Disorder	6	92	0.76	< 0.001
Schizophrenia	4	96	0.84	< 0.001
Enuresis	3	96	0.78	< 0.001
Separation Anxiety Disorder	3	96	0.78	< 0.001
Attention Deficit Hyperactivity Disorder	3	92	0.78	< 0.001
Conduct Disorder	3	96	0.78	< 0.001

### Diagnostic efficiency

The K-SADS-PL DSM-5 diagnoses showed sensitivities ranging from 0.93 to 1, with the lowest sensitivity found for major depressive disorder. Specificity values were consistently high (> 90%) across all diagnostic categories, except for major depressive disorder (84%). Positive predictive validity was moderate to low, ranging from 33 to 73% in all diagnostic categories except for major depressive disorder (92%). Notably, the high negative predictive validity values for all diagnostic categories indicate that there were no false negatives in any of the K-SADS-PL-GR-5 diagnoses, as shown in Table 3.

**Table 3:** Sensitivity, specificity, positive predictive value, and negative predictive value of the K-SADS-PL-GR-5

	PPV	NPV	Sensitivity	Specificity
Diagnosis	%	%	%	%
Major Depressive Disorder	92	85	93	84
Persistent Depressive Disorder	69	100	100	96
Attention Deficit Hyperactivity Disorder	50	100	100	96
Conduct Disorder	50	100	100	97
Generalized Anxiety Disorder	63	100	100	93
Separation Anxiety Disorder	50	100	100	98
Panic Disorder	33	100	100	98
Specific Phobia	67	100	100	99
Obsessive Compulsive Disorder	64	100	100	96
Schizophrenia	73	100	100	97
Enuresis	67	100	100	98

### Convergent and divergent validity

The K-SADS-PL DSM-5 and CDI total score demonstrated moderate to strong convergent validity coefficients. Strong and significant correlation, with large effect size (d = 1.53), was found between mood disorders as a spectrum and CDI (r = 0.57, p < 0.001). Major depressive disorder showed moderate correlation coefficient (r = 0.34, p < 0.05), with medium effect size (d = 0.72). Persistent depressive disorder showed a moderate correlation coefficient (r = 0.27, p < 0.01), with medium effect size (d = 0.72). However, convergent validity evidence for anxiety disorders was poor relative to the SCARED scale (Table 4). In terms of divergent validity, all mood disorders showed no significant correlation with the SCARED scale. Furthermore, anxiety disorders as a spectrum displayed poor correlation with the CDI scale, as shown in Table 4.

**Table 4:** Correlation and effect size between K-SADS-PL-GR-5, CDI and SCARED

		CDI		SCARED	
Diagnosis		r	Cohen’s d	r	Cohen’s d
Any mood disorder	(N = 59)	0.574***	1.528***	0.272	0.981
Major Depressive Disorder	(N = 48)	0.342*	0.722***	0.272	0.981
Persistent Depressive Disorder	(N = 16)	0.276**	0.719**	0.083	0.197
Any anxiety disorder	(N = 19)	0.227*	0.549*	0.364	0.921
Generalized Anxiety Disorder	(N = 14)	0.201	0.541*	0.349	0.723

CDI: Children’s Depression Inventory; SCARED: Screen for Child Anxiety Related Emotional Disorders

* p < 0.05

** p < 0.01

*** p < 0.001

## Discussion

The present study aimed to evaluate the psychometric properties of the K-SADS-PL DSM-5 version in a Greek sample, specifically examining inter-rater reliability, consensual validity, and construct validity. This is the first study investigating the reliability and validity of the K-SADS-PL DSM-5 version in Greece.

In line with previous studies on K-SADS-PL DSM-IV in other languages [5, 6, 10–12, 14], our findings revealed good to excellent inter-rater reliability for the Greek version of K-SADS-PL DSM-5. This is consistent with our previous study using the Greek version of the K-SADS-PL [18]. These findings support the applicability of the K-SADS-PL DSM-5 as a reliable diagnostic tool in Greek-speaking populations.

Consensual validity, evaluated by comparing K-SADS-PL DSM-5 diagnoses with clinical diagnoses, demonstrated good to excellent consensual validity across most psychiatric diagnoses, with the exception of panic disorder, which showed fair validity. These results are consistent with previous studies that have reported good consensual validity for the K-SADS-PL DSM-5 [14]. The higher prevalence of disorders in the Greek version of K-SADS-PL DSM-5 compared to clinical diagnoses may reflect the comprehensive assessment of symptomatology provided by the K-SADS-PL DSM-5, potentially leading to increased diagnostic accuracy. Diagnostic efficiency, measured by sensitivity, specificity, positive and negative predictive values, showed consistently high specificity and negative predictive validity across all diagnostic categories. However, positive predictive validity was found to be medium to low, except for major depressive disorder. These findings suggest that the K-SADS-PL DSM-5 may be more effective in ruling out psychiatric disorders than in confirming their presence, particularly in cases of comorbidity.

Construct validity was assessed by examining correlations between the Greek version of K-SADS-PL DSM-5 diagnoses and scores on the CDI and SCARED scales. Our results showed medium to strong convergent validity coefficients between the K-SADS-PL DSM-5 and the CDI for mood disorders, with large effect sizes. However, convergent validity evidence for anxiety disorders relative to the SCARED scale was found to be poor. These findings may be attributed to differences in the assessment methods used for anxiety disorders (K-SADS-PL-GR-5 interview versus SCARED self-report) or to differences in the specific anxiety disorders included in the K-SADS-PL DSM-5. In terms of divergent validity, mood disorders did not show significant correlations with the SCARED scale, and anxiety disorders displayed poor correlations with the CDI scale.

There are several limitations to the present study. First, the utilization of an unstructured clinical interview as a gold standard for validating the K-SADS-PL DSM-5 may have led to an unreliable estimate of diagnosis, due to subjective judgement and incomplete elicitation of diagnostic information. However, clinician’s judgment during all diagnostic procedures was complemented by additional use of available data sources (information provided from a caregiver and a teacher of the patient previous records, psychological assessments). Second, the study did not include test-retest reliability or assessment of the screen criteria for the K-SADS-PL DSM-5, which could provide further evidence for its reliability and validity. Finally, the small number of cases with clinical diagnoses provided allowed to establish only 11 Kappa values, might support limited information on consensual validity of the K-SADS-PL DSM-5.

Despite these limitations, our findings provide initial support for the psychometric properties of the Greek version of the K-SADS-PL DSM-5. The good to excellent inter-rater reliability, consensual validity, and construct validity suggest that the Greek version of K-SADS-PL DSM-5 may be a useful diagnostic tool in Greek-speaking populations. Further research is needed to examine the test-retest reliability, screen criteria, and psychometric properties of the Greek version of K-SADS-PL DSM-5 in larger, more diverse samples and various clinical settings. Moreover, future studies should explore the utility of the K-SADS-PL DSM-5 in detecting psychiatric comorbidities and predicting treatment outcomes.

In conclusion, the present study provides preliminary evidence supporting the use of the K-SADS-PL DSM-5 as a reliable and valid diagnostic tool for assessing psychiatric disorders in Greek-speaking populations. Our findings contribute to the growing body of literature on the psychometric properties of the K-SADS-PL DSM-5 across different languages and cultural contexts. By validating the K-SADS-PL DSM-5 in the Greek context, we hope to improve the early identification and accurate diagnosis of psychiatric disorders in Greek-speaking children and adolescents, ultimately leading to more effective treatment and better outcomes.

## Data Availability

The datasets used and/or analyzed during the current study are available from the corresponding author on reasonable request.

## References

[1] Kaufman J, Birmaher B, Brent D, Rao U, Flynn C, Moreci P (1997). Schedule for affective Disorders and Schizophrenia for School-Age Children-Present and Lifetime Version (K-SADS-PL): initial reliability and Validity Data. J Am Acad Child Adolesc Psychiatry.

[2] American Psychiatric Association. (1994). Diagnostic and statistical manual of mental disorders (4th ed.).

[3] American Psychiatric Association. (2013). Diagnostic and statistical manual of mental disorders (5th ed.). 10.1176/appi.books.9780890425596.

[4] Kaufman J, Birmaher B, Axelson D et al. Schedule for affective Disorders and Schizophrenia for School-Aged children: Present and Lifetime Version (K-SADS-PL) DSM-5 November 2016 working draft. New Haven, Yale University, Child and Adolescent Research and Education.

[5] Ulloa RE, Ortiz S, Higuera F, Nogales I, Fresán A, Apiquian R, Cortés J, Arechavaleta B, Foulliux C, Martínez P, Hernández L, Domínguez E, de la Peña F (2006). [Interrater reliability of the spanish version of schedule for affective Disorders and Schizophrenia for School-Age Children—Present and Lifetime version (K-SADS-PL)]. Act Esp Psiquiatr.

[6] Shanee N, Apter A, Weizman A (1997). Psychometric properties of the K-SADS-PL in an israeli adolescent clinical population. Isr J Psychiatry Relat Sci.

[7] Kim YS, Cheon KA, Kim BN, Chang SA, Yoo HJ, Kim JW, Cho SC, Seo DH, Bae MO, So YK, Noh JS, Koh YJ, McBurnett K, Leventhal B (2004). The reliability and validity of kiddie-schedule for affective Disorders and Schizophrenia-Present and Lifetime Version-Korean version (K-SADS-PL-K). Yonsei Med J.

[8] Brasil HHA, Bordin IA (2010). Convergent validity of K-SADS-PL by comparison with CBCL in a portuguese speaking outpatient population. BMC Psychiatry.

[9] Lauth B, Arnkelsson GB, Magnússon P, Skarphéðinsson G, Ferrari P, Pétursson H (2010). Validity of K-SADS-PL (schedule for affective Disorders and Schizophrenia for School-Age Children—Present and Lifetime Version) depression diagnoses in an adolescent clinical population. Nordic J Psychiatry.

[10] Marques CC, Matos AP, do, Céu Salvador M, Arnarson E, Craighead WE. Reliability and Validity of the Schedule for Affective Disorders and Schizophrenia for School-Age Children-Present and Lifetime Version (K-SADS-PL): Portuguese Version. Child Psychiatry Human Development. 2022; 53(6); 1119–1128. 10.1007/s10578-021-01196-5. 10.1007/s10578-021-01196-5 34050391

[11] Ünal F, Öktem F, Çetin Çuhadaroğlu F, Çengel Kültür SE, Akdemir D, Foto Özdemir D, Çak HT, Ünal D, Tıraş K, Aslan C, Kalaycı BM, Aydos BS, Kütük F, Taşyürek E, Karaokur R, Karabucak B, Karakök B, Karaer Y, Artık A (2019). [Reliability and validity of the schedule for affective Disorders and Schizophrenia for School-Age Children-Present and Lifetime Version, DSM-5 November 2016-Turkish adaptation (K-SADS-PL-DSM-5-T)]. Turk Psikiyatri Dergisi = Turk J Psychiatry.

[12] Þórðarson Ó, Ævarsson F, Helgadóttir S, Lauth B, Wessman I, Sigurjónsdóttir S, Smárason O, Harðardóttir H, Skarphedinsson G (2020). Icelandic translation and reliability data on the DSM-5 version of the schedule for affective disorders and schizophrenia for school-aged children – present and lifetime version (K-SADS-PL). Nord J Psychiatry.

[13] de la Peña FR, Villavicencio LR, Palacio JD, Félix FJ, Larraguibel M, Viola L (2018). Validity and reliability of the kiddie schedule for affective disorders and schizophrenia present and lifetime version DSM-5 (K-SADS-PL-5) spanish version. BMC Psychiatry.

[14] Nishiyama T, Sumi S, Watanabe H, Suzuki F, Kuru Y, Shiino T, Kimura T, Wang C, Lin Y, Ichiyanagi M, Hirai K (2020). The Kiddie schedule for affective Disorders and Schizophrenia Present and Lifetime Version (K-SADS-PL) for DSM-5: a validation for neurodevelopmental disorders in japanese outpatients. Compr Psychiatry.

[15] Kariuki SM, Newton CRJC, Abubakar A, Bitta MA, Odhiambo R, Phillips Owen J (2020). Evaluation of Psychometric Properties and Factorial structure of ADHD Module of K-SADS-PL in Children from Rural Kenya. J Atten Disord.

[16] Gindt M, Richez A, Battista M, Fabre R, Thümmler S, Fernandez A (2021). Validation of the French Version of the child posttraumatic stress checklist in French School-Aged children. Front Psychiatry.

[17] Boudjerida A, Labelle R, Bergeron L, Berthiaume C, Guilé J-M, Breton J-J (2022). Development and initial validation of the disruptive Mood Dysregulation Disorder Questionnaire among Adolescents from Clinic Settings. Front Psychiatry.

[18] Kolaitis G, Korpa T, Kolvin I, Tsiantis J (2003). Schedule for affective disorders and schizophrenia for school-age children-present episode (K-SADS-P): a pilot inter-rater reliability study for greek children and adolescents. Eur Psychiatry.

[19] Wild D, Grove A, Martin M, Eremenco S, McElroy S, Verjee-Lorenz A (2005). Principles of good practice for the translation and cultural adaptation process for patient-reported outcomes (PRO) measures: report of the ISPOR Task Force for Translation and Cultural Adaptation. Value in Health.

[20] Kovacs M, Children’s Depression. Inventory.1978; 10.1037/t00788-000.

[21] Giannakopoulos G, Kazantzi M, Dimitrakaki C, Tsiantis J, Kolaitis G, Tountas Y (2009). Screening for children’s depression symptoms in Greece: the use of the children’s Depression Inventory in a nation-wide school-based sample. Eur Child Adolesc Psychiatry.

[22] Birmaher B, Khetarpal S, Brent D, Cully M, Balach L, Kaufman J, Neer S (1997). The screen for child anxiety related Emotional Disorders (SCARED): scale construction and psychometric characteristics. J Am Acad Child Adolesc Psychiatry.

[23] Landis JR, Koch GG (1977). The measurement of observer agreement for categorical data. Biometrics.

[24] Cohen J. The effect size. Statistical Power Analysis for the Behavioral Sciences.1988: 77–83.

